# Culture and Professional Success

**Published:** 1893-01

**Authors:** 


### Culture and Professional Success.

In the *London Lancet*, September 17, 1892, the editor comments on a paper on this subject appearing in the September number of the *New Review* from the pen of the late Sir Morell Mackenzie. A strong plea is made for the advantages of culture in the classics of ancient and modern literature for the medical practitioner.

All educational methods are being subjected to the most stringent scrutiny, and the medical profession has been, and still is, engaged in revising its curricula and extending its period of study. Hence, an article like that cited, raising the great question how far general or "liberal" culture, as contrasted with special, technical, and professional education, is conducive to professional efficiency and success, is worthy of careful attention. The article is strongly in favor of a wide general culture as the best preparation for the more special training which every profession demands from those who aspire to enter it. The author's grounds are various, but they may be summed up under two heads: first, that such culture confers breadth and flexibility of mind and renders the acquisition of special knowledge easy; and, secondly, that it develops a knowledge of human nature, and hence assists in enabling us to treat our clients or patients, not as mere "cases," but as complex, sensitive and highly organized men and women.

This issue, ancient as it is, appears likely again to be fought out in our own day. There is a strong utilitarian wave of opinion now prevalent, and it is becoming generally assumed as an axiom that all training for a profession should be directed to the special needs and work of that profession, and that liberal studies, however pleasant or refining, must, in the stern struggle for existence, make way for subjects more practical and more immediately and obviously useful. From these causes arise the sacrifice of Greek, the limited study of Latin, the ignoring of English literature, and, on the other hand, the concentration of attention upon physical and biological science.

We are no advocates of any abortive and anachronistic attempt to turn medical students into classical or literary pedants as a preparation for the work of the hospital ward or the consulting-room; but we would remind those most imbued with the spirit of the new era, that the preparation of the intellect for the reception of knowledge is an object hardly less great and urgent than the character of the knowledge which is to be imparted.

We all admit that some such preparation is indispensable. The greatest enthusiast for science will hardly affirm that chemistry, physics, and biology afford a sufficient basis for an all-round intellectual development. They are no doubt most important, but their successful prosecution would still leave the student ignorant of the history of his race and of many of the highest and noblest achievements of the human intellect. In medical education we desire, with as little waste of time as possible, to bring trained and disciplined minds to bear on the problems of disease; and the question is, in what manner this can be best effected.

Many will accept this putting of the case, and will ask with derision how the differential calculus, the subtleties of Greek grammar, or the Categories of Kant are likely to be helpful. A sneer of this kind, however rhetorically effective, is not conclusive. The medical student needs strength and grasp of mind; the capacity to note resemblances and differences; the power of determining what amount of evidence is sufficient to warrant a given conclusion; some capacity of reading character, so as to estimate correctly a patient's statements and to eliminate the personal equation. He needs to be cautioned against haste, inaccuracy, prejudice and preconceived opinions, fallacious reasoning, confused or contradictory ideas, want of tact, and many other things.

These are formidable requirements, and the question is whether they can be met by any process less tedious and troublesome than that involved in a good preliminary education. The great danger of medical education at the present day is that raw youths with wholly unformed minds and destitute of any correct ideas regarding observation, evidence, and reasoning, should be set to dissect snails, muscles, and frogs, to look down microscopes, and to handle test-tubes, and to imagine that little else is necessary. If such methods should extensively prevail, we believe the results would be quite as disastrous as those which flow from an excessive devotion to the Greek digamma or the higher geometry. Cardinal Newman says in one of his works, that we have ample proof that the old methods of instruction by means of the classical languages and mathematical studies are capable of producing flexibility and grasp of mind, and that we have no proof that any other methods are adequate for this purpose.

Apart from purely professional work, we have to consider how best we can promote that mental attitude which helps the practitioner to understand his patient not only as the subject of disease, but as a human being. In the article alluded to above, it is justly observed that professional success depends not only upon the power of giving good advice, but upon the capacity of so influencing our patients that they will be inclined to follow that advice when given. Many of the greatest practitioners of medicine have a magnetic influence in this way; but for those not thus exceptionally endowed, there is no means of acquiring this power so likely to be successful as a wide knowledge of different subjects—in other words, a good all-round culture. This should enable its possessor to understand different types of mind, to enter into their point of view, and to sympathize with their various difficulties and apprehensions.—*Medical Age.*

